# Supplementary material for: Cryo-Treatment Enhances the Embryogenicity of Mature Somatic Embryos via the lncRNA–miRNA–mRNA Network in White Spruce
Source: Int J Mol Sci. 2022 Jan 20;23(3):1111. doi: 10.3390/ijms23031111 (PMC8834816; doi:10.3390/ijms23031111)
Supplement: Supplementary file 1 [file ijms-23-01111-s001.zip › ijms-1533166-supplementary.pdf]

Table S1. Primers for qPCR analyzed as reference genes.

Gene	Description	Forward/Reverse primer (5'-3')	References
PaEF1	*Picea abies elongation factor 1-α*	CACCTTGGGAGTGAAGCAAATG / CGGAGTAGTGGCATCCATCTTG	Vestman *et al*., 2011
PsACTIN	*Pinus sylvestris actin*	GGACAGGTCATTACCGTTGG / GATACCCGCTGCTTCCATT	Vuosku *et al*., 2009
PsUBQ	*Pinus sylvestris ubiquitin*	GAAGGAGCAGTGGAGTCCTG / CAATTTCAGGGACGAGAGGA	Alakarppa *et al*., 2018
Pa5S	*Picea abies* 5S ribosomal RNA	CAACCCATCCCGAACTTGG / Universal miRNA qPCR primer	Yakovlev *et al*., 2010
PatRNA-H1	*Picea abies* transfer RNA-His	TGATCCTGTGGCGATTTTGCAT / Universal miRNA qPCR primer	Yakovlev *et al*., 2010
PatRNA-R1	*Picea abies* transfer RNA-Arg	AACCACGGTGTCGGGGGTTC / Universal miRNA qPCR primer	Yakovlev *et al*., 2010

Table S2. Primers for the verification of mRNA.

Gene	Forward primer (5'-3')	Reverse primer (5'-3')	GenBank No.
WRKY7	AACACCTTGTACCGTCCCAC	ATGGCCTGGAACCCATGAAG	OK649318
WIN1	TGGTGGAGGCAGAAATACGG	TACGAGAAATCTACGCACGC	OK649319
LAX3	TGCAACAGTGGGCTATGTGAT	AGGTAGAAGCAGGCAACACT	OK649316
MYBS3	ATTAAGCCAAGTGCGCCAAC	TTGAGAGTGTTGTGAGGCGG	OK649317
GASA5	CATATCGGTGTTCGGCCACT	CCAGTTGTTGTAGCAAGGGC	OK649320
GST	GTTCGCATAGCACTTGCCCT	GTGTCCCATGCCTCCTCTATG	OK649315

Table S3. Primers for the verification of lncRNA.

Gene	Forward primer (5'-3')	Reverse primer (5'-3')
MSTRG.33602.1	TCATTGTGCTCTGCTCGACT	CTGAGTCTTATCCTGCCCACC
MSTRG.505746.1	ACACCAACCTCTCTTGTTGAA	ACAACTACACGACGGAGCAG
MSTRG.1070680.1	GAGCGATGGAAGGAGAAACAAC	TTGAAAGGGACGATCCACACAG

Table S4. Primers for the verification of miRNA.

Gene	Forward primer (5'-3')
Novel_miR_339	AGGATATGAAGATTTGGTGGA
Novel_miR_495	ATGGTCAAGATGTTTAATGAA
Novel_miR_527	TCTATGATTGAAGTTTTGG
